# Necrotizing Gingivitis: Microbial Diversity and Quantification of Protein Secretion in Necrotizing Gingivitis

**DOI:** 10.3390/antibiotics10101197

**Published:** 2021-10-01

**Authors:** Nicolas Gerhard, Thomas Thurnheer, Susanne Kreutzer, Rudolf Dominik Gmür, Thomas Attin, Giancarlo Russo, Lamprini Karygianni

**Affiliations:** 1 Clinic for Conservative and Preventive Dentistry, Center of Dental Medicine, University of Zurich, Plattenstrasse 11, 8032 Zurich, Switzerland; nicigerhard@bluewin.ch (N.G.); Thomas.Thurnheer@zzm.uzh.ch (T.T.); nickgmuer@bluewin.ch (R.D.G.); Thomas.Attin@zzm.uzh.ch (T.A.); 2 Functional Genomics Center Zurich, University of Zurich/ETH Zurich, 8057 Zurich, Switzerland; susanne.kreutzer@fgcz.ethz.ch (S.K.); giancarlo.russo@uzh.ch (G.R.)

**Keywords:** fluorescence in situ hybridization (FISH), 16s rRNA, microbial metagenome, necrotizing gingivitis, multiplex bead array assays (MBAA), cytokines

## Abstract

Necrotizing gingivitis (NG) is a necrotizing periodontal disease that differs from chronic gingivitis (CG). To date, both the microbiological causes and the involved host cytokine response of NG still remain unclear. Here, we investigated corresponding interdental plaque and serum samples from two groups of Chinese patients with CG (n = 21) or NG (n = 21). The microbiota were studied by 16S rRNA Illumina MiSeq sequencing of the microbial metagenome and by assessing quantitatively the abundance of the phylum *Bacteroidetes*, the genus *Prevotella* and the species *T. forsythia*, *P. endodontalis*, and *P. gingivalis* using fluorescence in situ hybridization (FISH). With respect to the associated host response, the levels of 30 inflammatory mediators were quantified by multiplex immunoassay analysis. Differential microbial abundance analysis of the two disease groups revealed at the phylum level that *Proteobacteria* accounted for 67% of the differentially abundant organisms, followed by organisms of *Firmicutes* (21%) and *Actinobacteria* (9%). At the species level, significant differences in abundance were seen for 75 species of which 58 species were significantly more abundant in CG patients. Notably, the FISH analysis revealed that *Bacteroidetes* was the most prevalent phylum in NG. The multiplex cytokine assay showed significant quantitative differences between the disease groups for eight analytes (GM–CSF, G–CSF, IFN–α, IL–4, IL–13, TNF–α, MIG, and HGF). The G–CSF was found to be the most significantly increased inflammatory protein marker in NG. The next-generation sequencing (NGS) data supported the understanding of NG as a multi-microbial infection with distinct differences to CG in regard to the microbial composition.

## 1. Introduction

Necrotizing gingivitis (NG) is a necrotizing periodontal disease with the characteristic presentation of an acute, painful, and destructive process. The progression of the disease can lead to necrotizing stomatitis and noma [1]. Characteristic clinical findings define the diagnosis of NG: gingival pain; interdental necrosis, which appears as punched-out gingival papilla; and gingival bleeding [2]. Further NG-related features are foetid breath, pseudomembrane formation, and extraoral lymphadenopathy [3,4]. Originally described as necrotizing ulcerative gingivitis, the term “ulcerative” was later eliminated, because ulceration was considered to be secondary to necrosis [4].

Early findings regarding the NG-associated microbial composition of dental plaque indicated the predominance of an endogenous, opportunistic fusiforme-spirochetal infection [5,6,7,8,9]. However, further studies have demonstrated that NG is a more complex and variable mixed microbial infection, which does not necessarily include the predominance of a fusiforme-spirochetal complex as prescribed by Plaut and Vincent [2,10,11,12]. In the meantime, *Prevotella intermedia*, *Selenomonas* spp., and *Peptostreptococcus* spp. are also considered to be highly NG-related bacterial species [3]. The NG-related gingival infection can be modified by particular risk factors and interacts with a variety of host factors without a predominant periodontal pathogen [13,14].

Depending on the population, the prevalence of NG varies broadly [15], although it is generally rather low (<1%), especially in the industrialized societies of North America, Europe, and Japan. Most likely, this is due to high health standards in these countries [13]. Interestingly, the true prevalence of NG has to be further investigated due to the fact that relevant epidemiologic data were mainly obtained from non-representative population groups (low socioeconomic class [16], HIV patients [17], military officers and soldiers [18], and urban slum residents [13,19]) providing a probably skewed estimation.

In contrast to NG, chronic gingivitis (CG) is a widespread phenomenon, mainly in children and adolescents [20]. Although the microbial etiology of CG is proven [21,22] and similar to NG, the link to a specific individual or group of microorganisms has not been proven [23,24].

According to the new classification scheme for periodontal diseases [3,25], CG is classified as a gingival disease, whereas NG constitutes a subgroup of periodontitis. Findings have concluded that CG is a reversible state and a necessary prerequisite for periodontitis, which is irreversible and more severe [26]. Therefore, patients with treated and stable periodontitis are at higher risk for recurrent periodontitis than CG patients [26]. The clinical symptoms of both diseases (CG, NG) vary broadly; the term CG describes the inflamed state of the gingiva with >10% bleeding sites [27,28], and, upon clinical examination of bleeding on probing (BOP %), probing depths > 3 mm [29]. Necrotizing diseases like NG show three typical clinical features: pain, bleeding, and ulceration of the gingival interdental papilla [3]. The extent, severity, and progression of CG and NG are mainly dependent on host-related factors and systemic modifying factors [3,26].

Cytokine levels in NG samples have not been investigated so far. Although there have been extensive studies on inflammation markers in CG and periodontitis in general [30], which have confirmed elevated levels of specific cytokines depending on the clinical severity of the disease, there are no literature reports describing the immunological status of NG patients [31].

Due to the fact that only limited studies have investigated samples of NG-infected patient groups so far, little is known about the microbial composition of dental plaque and expression of inflammation markers in NG. To date, some bacterial species (*Porphyromonas gingivalis, Actinomyces gerencseriae, Prevotella intermedia, Prevotella nigrescens* [13]) have been detected using fluorescence in situ hybridization (FISH) or immunofluorescence (IF), whereas the presence of other putatively pathogenic bacterial groups in NG (*Tannerella forsythia, Porphyromonas endodontalis,* phylum *Bacteroidetes*, families *Bacteroidaceae* and *Prevotellaceae*) has not yet been ascertained.

The objective of this study is to profile the immunologic as well as the microbial landscapes associated with NG and CG and to detect differences that might help elucidate NG- and CG-specific inflammatory and bacterial markers.

## 2. Material and Methods

The study protocol was approved by the Ethics Committee of the University of Zurich (Basec Nr. Req-2019–01260). All assay protocols and data sampling were conducted in accordance with relevant institutional and national guidelines and regulations. Thirty inflammatory proteins were tested using an ELISA assay based on blood serum samples, while marginal plaque was chosen to quantify six bacterial probes using FISH and fluorescence microscopy and to extract DNA for 16S rRNA amplicon sequencing. The cohort consists of 42 individuals, 21 NG, and 21 CG patients.

### 2.1. Patients and Sample Collection

Patient selection, clinical examination, and collection of interdental marginal plaque took place in 1998/1999 and were described previously [13,32]. In brief, upon giving their written informed consent, 42 otherwise healthy Chinese patients with either NG (n = 21; mean age of 36.9 ± 6.7 years) or CG (n = 21, 38.9 ± 6.3 years) formed our test groups. All NG patients had multiple typical clinical signs for NG, such as interdental necrosis, characterized by the loss of gingival papillae, pseudomembrane formation, fetid odor, gingival pain, and ulceration [2], whereas CG patients displayed strong gingival inflammation in the absence of any of the mentioned characteristic signs of NG. Marginal supragingival plaque samples were collected from the buccal and/or oral surfaces of the most disease-affected regions following a procedure previously described by the authors [13,32,33,34,35,36,37]. Sample material from three infected sites was pooled in 1 mL reduced transport fluid (RTF) containing 10% glycerol [38]. Plaque samples were split into aliquots and stored in liquid nitrogen.

Peripheral blood samples were taken from the median cubital vein and the serum was stored at –80 °C. Exclusion criteria during patient selection were (i) presence of any form of severe systemic disease or diseases of the salivary glands, (ii) dentition with less than 20 teeth, (iii) periodontal pockets with probing pocket depth 4 mm, and (iv) use of antibiotics or local antimicrobial mouth rinses such as chlorhexidine (CHX) within the last 3 months.

### 2.2. DNA Extraction

The DNA from plaque samples (NG, CG) was isolated using the GenEluate bacterial genomic DNA kit (Sigma-Aldrich, Saint Louis, MI, USA). The manufacturer’s recommendations were followed except for slight modifications to the pretreatment steps required for the lysis of Gram-positive bacteria: we extended lysis to 1 h using a mix of lysozyme (2.115 × 10^6^ U/mL), mutanolysin (250 U/mL), lysostaphin (200 U/mL) and achromopeptidase (600–1200 U/mL)) followed by treatment with proteinase K for 20 min. The extracted DNA was eluted twice in 60 µl preheated elution buffer. The amount of isolated DNA was determined using a NanoDrop ND-1000 spectrophotometer (Thermo Fisher Scientific, Waltham, MA, USA).

### 2.3. Amplification and Illumina MiSeq High-Throughput Sequencing

The variable regions V1–V3 of the 16S rRNA gene were amplified by PCR using primers 27F 5- GAG TTT GAT CCT GGC TCA GAT TGA ACG C-3 and 534R 5-XXXXXXXX ATT ACC GCG GCT GCT GG-3 [39]. PCR was carried out in a total volume of 50 µL containing 1× NEBNext High-Fidelity PCR Mastermix (New England BioLabs), 1.25 µM of each primer, and 5 µl of sample DNA. PCR steps included initial denaturation at 98 °C for 30 s, followed by 30 cycles consisting of denaturation at 98 °C for 10 s, annealing at 65 °C for 30 s and extension at 72 °C for 30 s and a final extension step at 72 °C for 5 min. The PCR products were purified with the NucleoSpin Gel and PCR Clean-up Kit (Macherey-Nagel, Düren, Germany) according to the manufacturer’s protocol, using a 1:4 dilution of the binding buffer NTI. The amount and the quality of the purified PCR amplicons were analyzed by a Qubit fluorometer (Invitrogen, Waltham, MA, USA,) and by agarose gel electrophoresis. For sequencing, the PCR amplicons were pooled in equimolar amounts.

The reverse primer contains inline barcodes for multiplexed sequencing. PCR products were pooled equimolar and Illumina sequencing adapters were attached using the NEB Next Ultra II Library preparation Kit (NEB, Ipswich, MA, USA). The samples were sequenced with 300 cycles paired-end on a MiSeq (600 cycles V3, Illumina, Inc, San Diego, CA, USA) and demultiplexed according to the inline barcodes.

### 2.4. Analysis of the 16S rRNA Data

The fast files generated by the MiSeq were quality-filtered and adapters-trimmed using Trimmomatic v0.39 [40]. Paired-reads that passed such preprocessing steps were joined using fastq-join [41] and then aligned to the Silva bacterial database using minimap2 [42]. Based on the alignments, genus-level abundances were obtained by aggregating all the species with the same parent genus and compared with the FISH data. Krona plots were generated using KronaTools [43].

### 2.5. Fluorescence In Situ Hybridization (FISH)

For semi-quantitative analysis of the phylum *Bacteroidetes* (CFB935), the families *Bacteroidaceae* and *Prevotellaceae* (BAC303), and the genus *Prevotella* (PRV392), as well as the bacterial species *Porphyromonas gingivalis* (L-Pgin1006–2)*, Porphyromonas endodontalis* (Pend740), and *Tannerella forsythia* (Tfor127), FISH was performed. In brief, the aliquots of the pooled marginal plaque samples were thawed, homogenized by the standard vortexing–sonication procedure for 10 s at 40–50 W on ice (Sonifier B-12; Branson, Danburg, CT, USA). The CG and NG samples were diluted (1:50) in coating buffer (0.9% NaCl, 0.02% NaN_3_, 2.5 × 10^–4^% hexadecyltrimethylammonium bromide; CTAB) [34]. Ten microliters of these suspensions were divided and fixed on 24-well epoxy-coated slides with a well diameter of 4 mm (Cell-Line, Erie Scientific Company, Portsmouth, NH, USA) to be used for FISH as previously described [34]. To include Gram-positive bacteria, additional permeabilization was conducted as follows: 2 min exposure to 7 μL/well of lysozyme (70 U/μL), aspiration of lysozyme droplet, brief dipping in nanopure water (H_2_O) followed by air-drying [44]. Blocking of unspecific binding included treatment with Denhardt’s solution (Sigma-Aldrich, St. Louis, MO, USA; diluted (1:50) in 0.9% NaCl) in the presence of protectRNA RNase inhibitor (1:500, Sigma-Aldrich Chemie GmbH, Buchs, Switzerland) in 0.9% NaCl and incubation for 30 min at 37 °C [33]. Seven validated, labeled at the 5′- end with Cy3 or carboxyfluorescein (FAM) (Microsynth, Balgach, Switzerland) and specific oligonucleotide rRNA-directed probes were used. The sequences of the rRNA directed probes and bacterial targets are listed in Table 1.

The universal probe EUB338 was used as a reference [47]. The final probe concentrations added to the individual wells were 5 ng µL^−1^ for Cy3 (Cyanine dye) and 20 ng µL^−1^ for FAM conjugates. Depending on the probe, 40–50% formamide as part of the hybridization buffer was used. The backbone of the hybridization process is represented by the previously described methods of Manz et al. [51] and Züger et al. [33], successively modified by Thurnheer et al. [52]. More precisely, the following alterations were made to the workflow described in [33]: (i) increased duration of hybridization (240 min), (ii) general DNA cell staining using 4′,6-diamidino-2-phenylindole (DAPI) incorporated in glycerol-based mounting fluid (VECTASHIELD^®^ Mounting Medium, Vector Laboratories Ltd., Peterborough, UK) was performed for all controls.

### 2.6. Image Acquisition and Analysis

As described in earlier studies [44], upon FISH, the stained bacteria were visualized using an Olympus BX60 epifluorescence microscope fitted with phase-contrast (Olympus Optical AG, Volketswil, Switzerland), an HBO 103 W/2 mercury photo optic lamp for excitation (OSRAM Lighting AG, Winterthur/Toess, Switzerland) and Olympus filter sets U-MNIBA (FAM, FITC; excitation, 470–490 nm; emission, 515–550 nm) and U-MA41007 (Cy3; excitation, 530–560 nm; emission, 575–645 nm). Additionally, the 4′,6-diamidino-2-phenylindole (DAPI) filter set U-MWU (excitation, 330–385 nm; emission > 420 nm) was used for DNA staining. Exposure times for Cy3 and FAM were set at 800 ms and 2000 ms, respectively. Phase contrast-, colored-, and grayscale images of the FISH samples were taken as 8-bit micrographs with an Olympus DP74 camera and the cellSens Entry 1.15 Imaging Software (Olympus Optical AG, Volketswil, Switzerland). At least 10 viewing fields per well at 1000× magnification were chosen with respect to: (i) absence of artifacts or aggregates, and (ii) presence of DAPI—stained bacteria. The grayscale images were further analyzed using cellSens Dimension Desktop 1.15 Imaging Software (Olympus Optical AG, Volketswil, Switzerland). For improved detection, automatic contouring of every micrograph was conducted. The covering grades of the stained bacteria (in %) were estimated by setting the areas of the Cy3 colored probes (target species) and FAM colored probe (total eubacteria) in relation to each other. The density threshold of the bacterial boundary was set manually for each image and stain. To be regarded as “true signals”, the objects had to be: (i) positive after using phase-contrast imaging and DAPI to avoid inclusion of possible false-positive signals, and (ii) positive for eubacteria. The areas considered to be positive for the respective probe were cross-checked by both phase-contrast- and colored images. The total area covered by the fluorescently labeled bacteria was described as an absolute value in µm^2^. The stained area with positive fluorescence signals for the EUB338 probe was taken as reference (100%) and the covering grade of the other probes was finally measured as a percentage (%) of the EUB338—positive areas.

### 2.7. Determination of Cytokines, Chemokines, and Growth Factors

The serum samples of the CG and NG patients were thawed and intensely vortexed for 30 s. Subsequent centrifugation at 4 °C at 21,000× *g* for 5 min in 1.5 mL Protein LoBind Tubes (Eppendorf AG, Hamburg, Germany) pelleted any insoluble material [53]. The final sample of 50 μL as duplicates (twice for a total of 100 μL) of clear supernatant was removed and used according to the instructions of the Human Cytokine Magnetic 30 Plex Panel (Novex, ThermoFisher Scientific, Waltham, MA, USA) [54]. This panel quantifies cytokines, chemokines, and growth factors in the collected medium [54]. Levels of the analytes in pg/ml were detected by multiplex bead array assays (MBAA) and read by Luminex^®^ 200 (Bio-Rad Laboratories Inc., Hercules, CA, USA). Analysis of the data was conducted by polynomial interpolation in Microsoft Excel for further statistical processing.

### 2.8. Statistical Analysis

To check for statistical differences between the two clinical groups with respect to the quantitative protein assay and FISH staining, a Mann–Whitney test was employed. We used Bonferroni (in the case of the FISH assay) or Benjamini–Hochberg correction (in the case of the ELISA assay, due to the presence of protein families) to account for multiple testing. In order to compare differential abundances between the groups in the 16S rRNA-based NGS data, edgeR [55] was used.

## 3. Results

### 3.1. Analysis of the Bacterial Communities in the Two Cohorts: FISH

**Figure 1:**
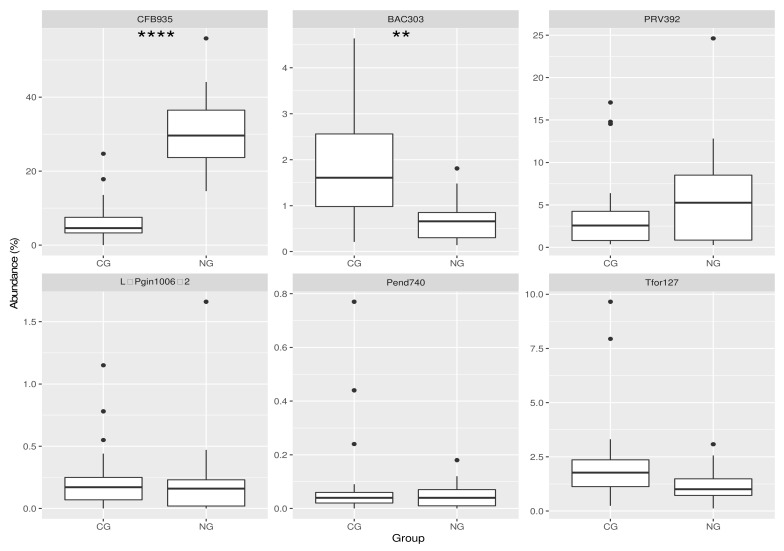
Box plots showing the abundance of six taxa in relation to the total amount of bacteria in samples from patients with chronic (n = 21) or necrotizing gingivitis (n = 21). Data are shown for the phylum *Bacteroidetes*, the families *Bacteroidaceae* and *Prevotellaceae*, the genus *Prevotella* and the species *P. gingivalis*, *P. endodontalis* und *T. forsythia*. Note that y-axis scales are different for each taxon. 16S rRNA probes used to detect the different taxa are described in Supplementary Table S1. *p*-values were calculated with the Bonferroni-corrected Mann–Whitney test; *p* < 0.0001 (****), *p* < 0.01 (**). CG, chronic gingivitis; NG, necrotizing gingivitis. CFB935; BAC303; PRV392; L–P GIN 1006–2; Pend740; Tfor127 are probes with different targets (see Table 1).

Box plots in Figure 1 depict the relative abundance of the six hybridized oligonucleotide probes in the FISH—treated dental plaque samples from subjects with CG and NG. The corresponding targets of the probes can be seen in Supplementary Table S1; CFB935 is considered as the “*Bacteroidetes*” probe.

Probe CFB935 represents, on average, the probe that, among those tested, captured the largest amount of microorganisms in both NG and CG biofilms. However, at 30.6% and 6.7%, there is a strong significant difference (*p* < 0.0001) between the two groups; in particular, the probe CFB935 was significantly more abundant in NG. Interestingly, among CG patients we identified one outlier, showing a high CFB935 abundance (24.7%), while in the NG group, two patients presented a lower proportion of cells stained by this probe (17.5% and 14.6%).

The other probe which shows a statistical difference in its abundance between the two groups (*p* < 0.01) is BAC303. Its fraction is much lower than CFB935, with mean values in the CG and NG groups of 1.91% and 0.70%, respectively.

No significant differences were detected between CG and NG groups with respect to the cell content stained with the other four probes (Supplementary Table S2).

Supplementary Figure S1 contains representative fluorescence microscopy (FM) images of the same samples generated with FISH following hybridization with the aforementioned probes.

Fluorescence microscopy (FM) panels illustrating the presence of specific phyla, genera, and bacterial species related to necrotizing gingivitis. Images were generated with FISH and FM following hybridization with the following oligonucleotide probes: broad-spectrum CFB935 staining cells of the phylum *Bacteroidetes* with diverse morphology (A); BAC303 staining differently sized aggregates of *Bacteroides* spp., *Prevotella* spp., and *Porphyromonas* spp. (B); PRV392 staining rod-shaped cells of *Prevotella* spp. (C); Pend740 and L-Pgin1006-2 staining small, round cells of *P. endodontalis* (D) and *P. gingivalis* (E), respectively. The bacterial targets are zoomed and highlighted on white-framed sub-images in panels A and C.

### 3.2. Analysis of the Bacterial Communities in the Two Cohorts: 16S rRNA NGS Comparison with FISH Data

**Figure 2:**
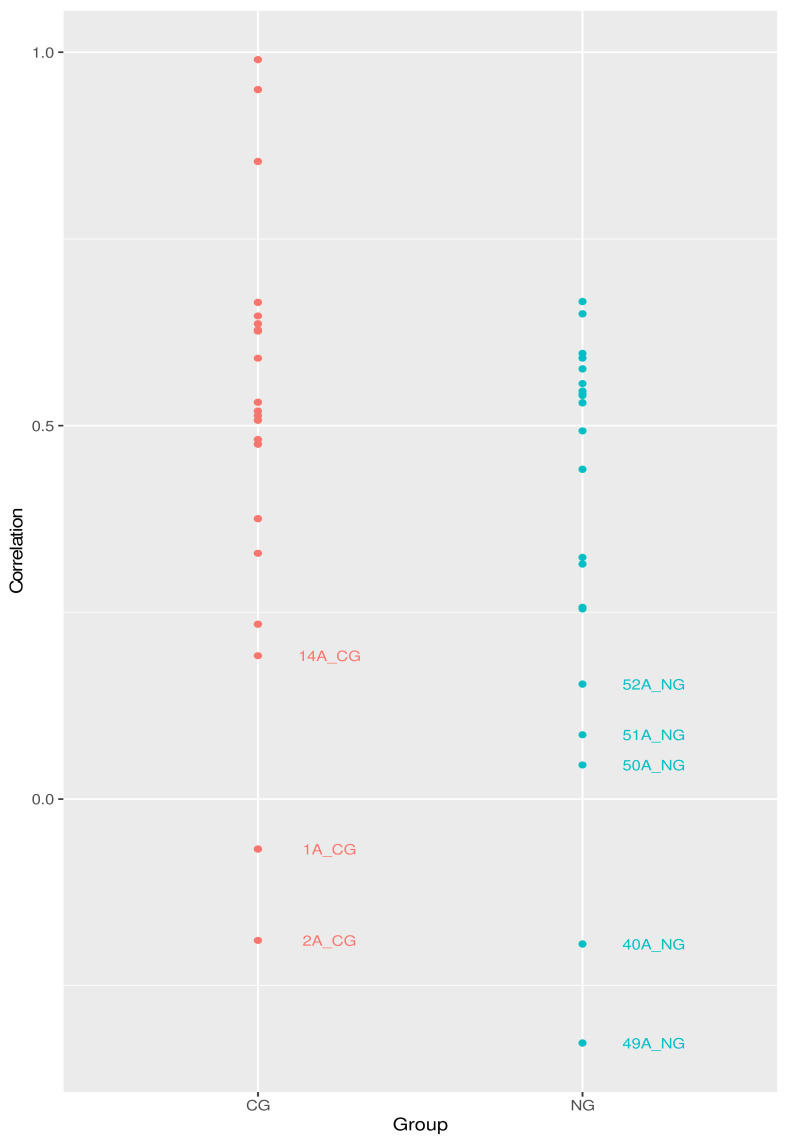
Correlation between the FISH-based and NGS-based relative abundances of the selected microbial species. The plotted value is the Pearson coefficient between the species quantities in each sample. Samples with R^2^ < 0.2 are labeled.

In addition to using FISH probes to capture predefined organisms, we also amplified and sequenced the 16S rRNA V1-V3 regions in order to screen the microbial community of the cohorts. To gauge the robustness of the NGS profiling, we first compared it with the FISH data. In Figure 2, we show the coefficients of correlation between the relative abundances calculated based on the NGS analysis and those based on the FISH staining. Of course, this is only possible for the genera (or set of genera) included in the FISH probes.

For the majority of the samples, the level of correlations is above 0.5 and can be considered moderately strong. The samples labeled in Figure 2 have a correlation coefficient below 0.2 (very weak) and therefore we decided to remove them from further analysis of the NGS data. Additionally, samples 47A_NG, 33A_NG, and 5A_CG have also been removed from downstream analyses involving NGS-based data, because more than 50% of the species identified in the pool were not observed (zero counts).

### 3.3. Further Profiling the Microbial Communities

**Figure 3:**
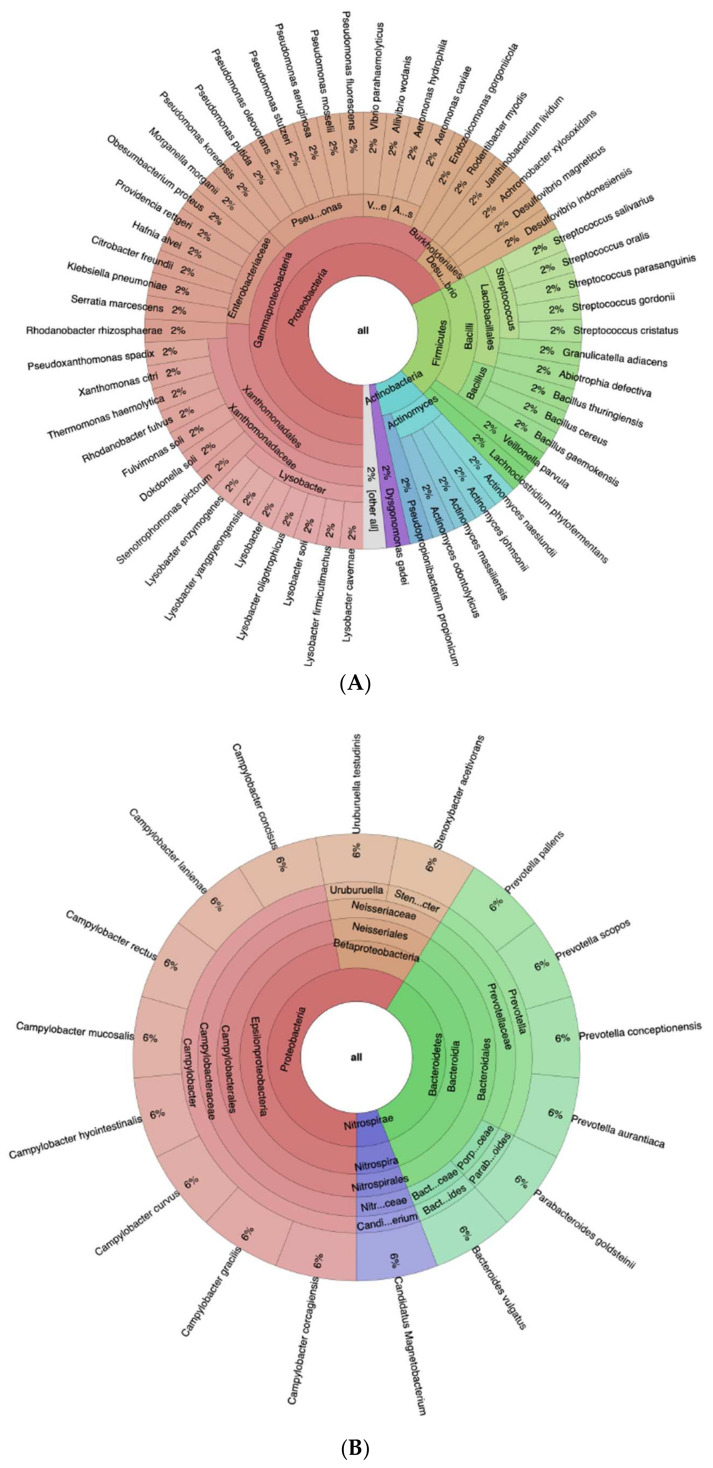
Krona plots of the taxonomies of the organisms which resulted in differentially abundant in the NGS analysis between the CG and NG groups. (**A**) Species overabundant in the CG group. (**B**) Species overabundant in the NG group. The full taxonomy of each species was reconstructed using the R package *myTAI* with the itis and NCBI databases.

A differential abundance analysis revealed 75 species as significantly different between the two groups, whose taxonomic distribution is depicted as Krona charts in Figure 3. Specifically, 58 species were significantly more abundant among CG patients, and the majority of such difference was driven by the phylum *Proteobacteria*, which accounted for 67% of the differentially abundant organisms, followed by *Firmicutes* (21%) and *Actinobacteria* (9%). Almost all of the *Proteobacteria* (90%) belonged to the class *Gammaproteobacteria*, which also accounted for two-thirds (60%) of the total number of differentially abundant species. *Pseudomonas* and *Lysobacter* are the most abundant genera (20% of Gammaproteobacteria, 12% of the total number of differentially abundant species), while almost half of the *Firmicutes* and 10% of the total number of differentially abundant species were *Streptococcus* spp.

Among the 17 species which were more abundant in the NG patients, 10 (59%) were *Proteobacteria*, of which 8 belonged to the *Campylobacter* family. The second most abundant phylum was *Bacteroidetes* (6 species, 35%); of those, 4 belonged to the *Prevotellaceae* family and one to the *Porphyromonadaceae*. This further confirmed the results obtained by FISH analysis with respect to the probe CFB935.

**Figure 4:**
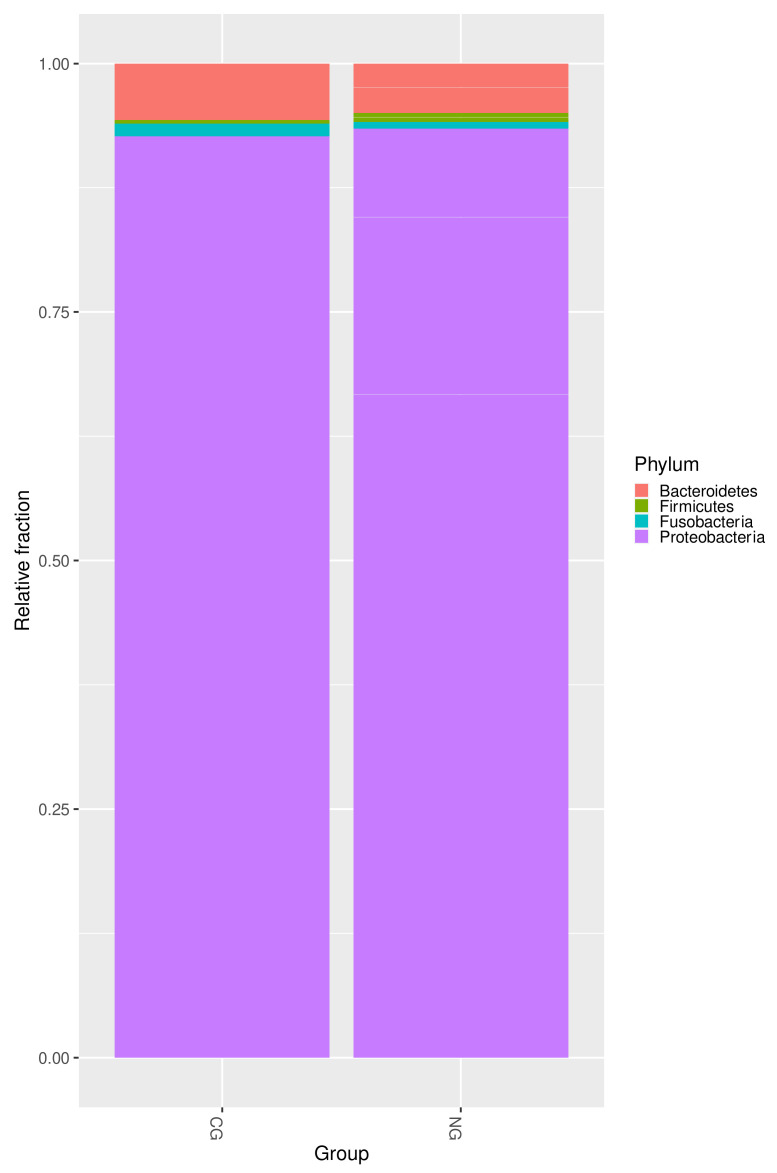
Relative prevalence of the ten most abundant species among the entire microbiome (“Full list”), the subset of species overabundant in the CG patients, and the subset of species overabundant in the NG.

A snapshot of the baseline microbial environment in the two groups is shown in Figure 4. Altogether, at the phylum level, the two groups had a very similar profile, with *Proteobacteria* being the most dominant phylum and representing ca. 90% of the population. However, as just discussed and shown in Figure 3, the deteriorating transition to NG was driven by a relative depletion of *Firmicutes* and a spike in *Bacteroidetes* to ca. 35%.

### 3.4. Analysis of the Expression of the Protein Markers

On average, the thirty proteins tested using the 30-plex MBAA displayed pretty similar expression levels between the groups (Supplementary Figure S2), including the very high concentration levels of RANTES (ca. 20 times the sample-wise mean in both NG and CG cohorts) and IL-8 (4 and 7 times the sample-wise mean in the NG and CG cohorts, respectively).

The heatmap shows the concentration (in pg/ml) of a total of 30 cytokines, chemokines and growth factors among chronic gingivitis and necrotizing gingivitis patients. The rows are ordered by mean concentration value stratified according to the color bar on the right.

**Figure 5:**
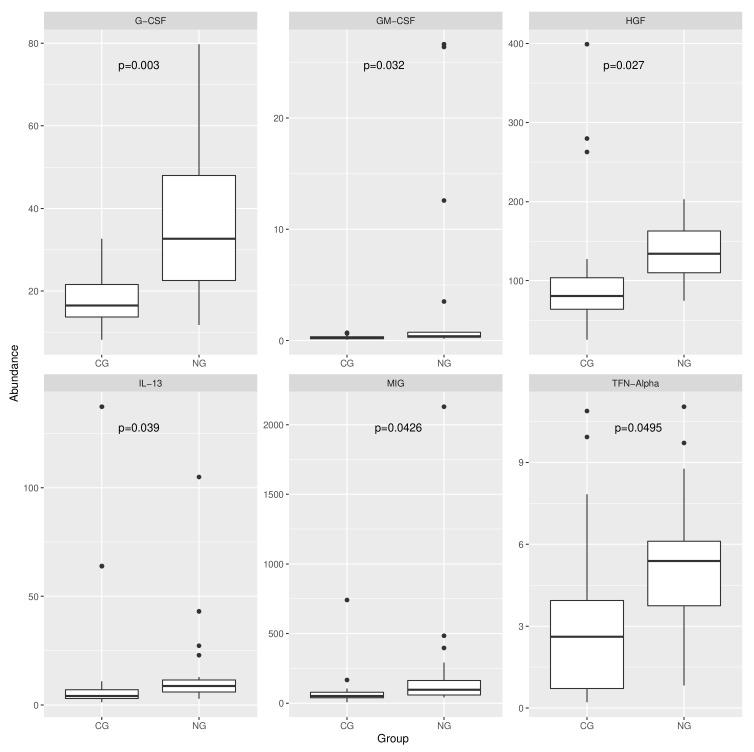
Box plots showing the concentration of the six proteins which are significantly differentially expressed between the NG and CG group. P-values were calculated using the Benjamini-Hochberg-corrected Mann-Whitney test.

Out of these 30 proteins, six (G-CSF, GM-CSF, HGF, IL-13, MIG, and TFN-α) were differentially abundant between the two groups (Figure 5). In all cases, the significantly higher protein expression was found in the NG cohort, suggesting a stronger inflammatory response driven by NG. However, it can be seen how in three cases (GM-CSF, IL-13, and MIG) the difference was driven by a handful of outliers over a rather low background signal, and therefore conclusions around these proteins should be more cautious. Of note, the cytokine G-CSF was the inflammatory marker with the highest level of up-regulation in necrotizing gingivitis, with a Benjamini–Hochberg adjusted *p*-value (*p* = 0.003) one order of magnitude more significant than the other five significantly different markers (Figure 5).

**Figure 6:**
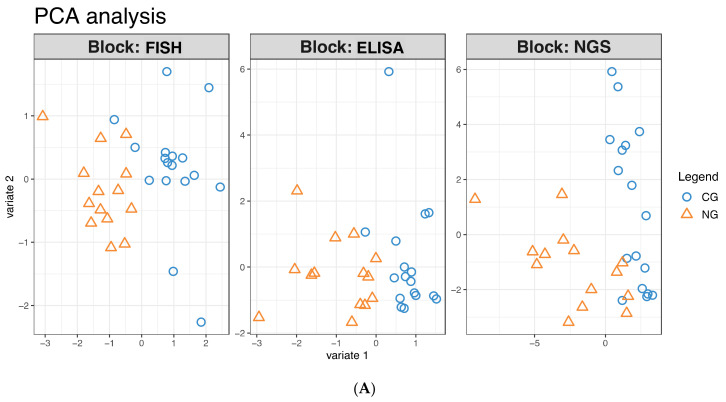

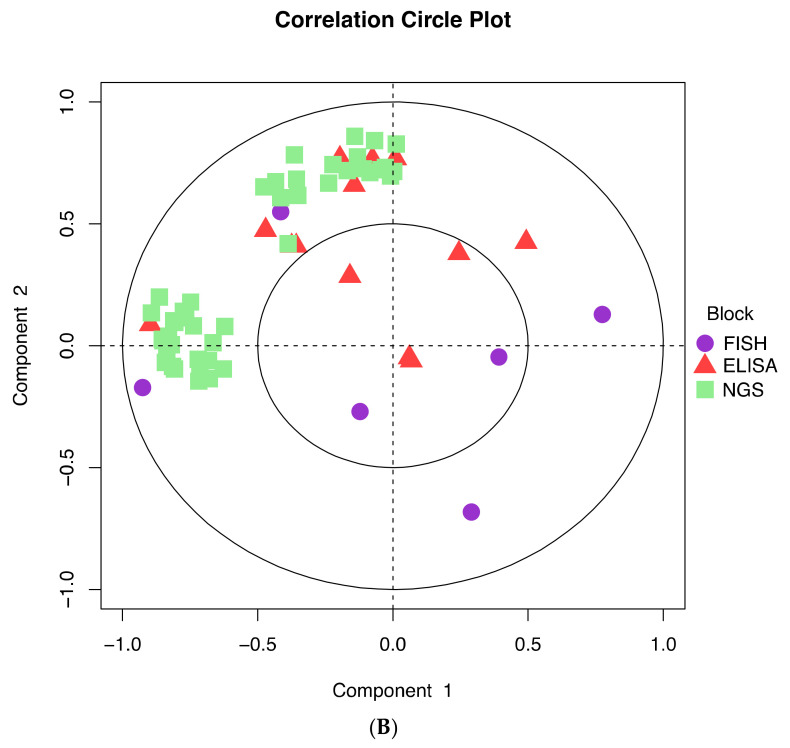

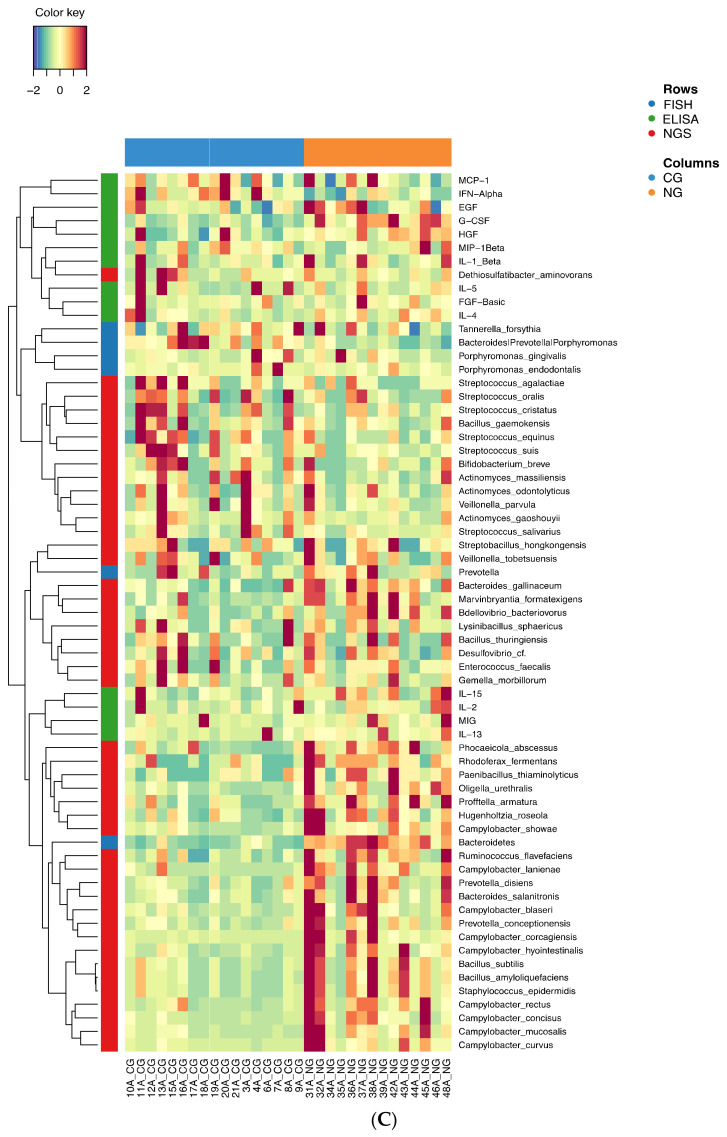
Integration of the three sources of data. (**A**) Individual principal component plots for the three blocks. Plot generated using the function plotIndiv from the package *mixOmics.* (**B**) Correlation circle plot: the further away from the origin, the higher the discriminative power of the features. Features clustering together represent potential co-signature from multiple data sources. Plot generated using the function plotVar from the package *mixOmics.* (**C**) Heatmap highlighting co-segregation of different data sources and the discriminative potential of the resulting clusters. The key color scales are based on Z-scores and scaled based on the row mean. Plot generated using the function cimDiablo from the package *mixOmics*.

With multiple sources of molecular information available, we also tried to investigate whether an interplay between the proteins and the microbial species associated with the diseases occurs. The three datasets (FISH, ELISA, and NGS) were integrated using the R package *mixOmics* with the main goal to identify co-signatures associated with multiple data sources (also called *blocks* in the package). As in each block, the main discriminative component was identified, and a correlation between such components was calculated for each pair of datasets. In this respect, the correlations were moderately strong for all three datasets, with the FISH and NGS blocks showing the highest correlation (R = 0.69). The corresponding discriminating effect of the individual blocks is shown in Figure 6A: one notices how robust discrimination between the groups was in all three blocks, which showed little to no overlap along the x-axis. However, the second component correlated well only in the NGS and ELISA datasets (R =0.59, Supplementary Table S3), suggesting a potential co-signature from these blocks.

Abundance and statistical analysis of the ELISA-based quantification for the 30 selected protein inflammatory markers.

Additional evidence that the NGS and ELISA blocks showed an additional predictive power and an interplay between some of their features is shown in Figure 6B: the main component, along the x-axis, was polarized by a cluster of mostly NGS features, whereas the second main component, along the y-axis, was polarized by another NGS-dominated cluster also including some ELISA features. Finally, we asked ourselves which features are actually driving this behavior, and the results are shown in Figure 6C. The dendrogram clearly splits the features into two main clusters, each of which branches into two further groups. For the bottom cluster, the top branch, starting with *S. hongkongensins* and ending with *D. magneticus* does not show any particular trend, while the bottom branch displays an overall enrichment in the CG group and comprises several *Streptococcus* spp. whose spike in the CG group is already shown in Figure 4. The top cluster is instead picking up the up-regulation in the NG group; the top branch includes the proteins (including two of the three—G-CSF, HGF—which were found differentially abundant by means of the ELISA assay). The bottom branch includes the NGS-identified species, clearly showing the many *Campylobacter* spp. which are overabundant in the NG group and essentially replicating the results observed by analyzing the NGS dataset alone in the classical way (Figure 3B). Such sub-clustering of protein and microbial features is another way to depict the interplay seen in the y-axis cluster in Figure 6B.

## 4. Discussion

The present study confirmed the assumption that NG is an opportunistic, mixed microbial infection. The FISH-stained microscopic images demonstrated the increased occurrence of the phylum *Bacteroidetes*, while the genera *Porphyromonas* spp. and *Tanerella* spp. showed an increase among NG patients (CFB935 probe). Several studies from our own group have so far focused on NG [13,32,33,34,35,36,37,56], revealing the prevalence of *Actinomyces gerencseriae*, *Campylobacter rectus*, *Fusobacterium nucleatum*/*Fusobacterium periodonticum*, *Porphyromonas gingivalis*, *Prevotella intermedia*/*Prevotella nigrescens*, and *Tannerella forsythia* after FISH-staining and subsequent FM [13,33,34,44,52,57]. The protein quantification revealed an increased expression of inflammatory markers in NG contrary to CG.

Plaque harboring highly diverse bacteria and the presence of the above-mentioned bacterial species has been proved by other 16S rRNA studies [58]. The 16S rRNA gene-based analysis is an effective method for the characterization of the oral microbiota [59]. The significantly higher prevalence of CFB935-stained oral genera in NG compared to CG patients confirmed the understanding of NG as a mixed microbial infection rather than a disease with abundant fusiform-spirochete bacterial flora [60]. The probe CFB935 described as Cytophaga–Flexibacter–Bacteroides assemblage contains among other species the putative periodontal pathogens *P. gingivalis*, *P. intermedia*, *P. endodontalis*, and *T. forsythia* [13,34,49,61,62]. In our study, about 30% of the bacteria stained with the EUB338 probe consisted of the aforementioned bacteria. The EUB338 probe detects most of the domain *Bacteria* except for target organisms of the phyla Planctomycetales and Verrucomicrobia [63]. These specific bacterial species may display an “inflammophilic” profile and can support the occurrence and progression of NG in the presence of a weak immune response [62]. However, the high incidence of *Bacteroidetes* in NG in contrast to CG may be attributed to the fact that the bacterial genome sequence data contain only part of the sequences of oral bacteria.

The high prevalence of *P. gingivalis* among NG patients (0.7%) versus CG patients (0.1%) was highlighted by Gmür et al. [13]. The results of the present study, which showed no significant differences in *P. gingivalis* between NG (0.2%) and CG (0.2%) samples, suggest that further studies on single bacterial species will be required in order to detect differences in the bacterial composition between NG and CG patients. A possible explanation for the non-significance could be the expression of different subtypes of fimA genotypes [64,65]. Additionally, it is possible that aggregates of diverse *Porphyromonas* are not appropriately represented in the image acquisition.

To have an overview of the microbial populations in the oral cavities of the patients, we amplified the 16S rRNA region from the DNA and performed high-throughput sequencing. On the one hand, the previously reported roles of individual species in the progression of NG was confirmed and expanded to close relatives, as in the case of *Campylobacter* and *Prevotella* spp. Both genera are known to be associated with periodontal disease [66] and our findings might point to their stronger involvement in the more advanced forms of pathologies such as NG. On the other hand, *Lysobacter* spp. and *Streptococcus* spp. were significantly upregulated in the CG samples. As *Lysobacter* spp. and *Streptococcus* spp. populate the oral cavity in normal conditions, and since gingivitis represents an early stage, less inflammatory form of periodontitis, it is likely that what we are observing is a displacement of some of these more common, generally innocuous species by their more pathogenic and inflammatory counterparts.

The immune response of a patient is modulated by proteins such as chemokines (chemotactic cytokines), cytokines, and growth factors [67,68,69,70], whose complex interaction leads to a vicious cycle with cumulation in tissue destruction and disease progression. The MBAA revealed that NG elicited the strong response of three serum cytokines (G-CSF, TNF-α, and HGF) and an additional, albeit less conclusive, upregulation of further three cytokines (GM-CSF, IL-13, and MIG).

The NG is a severe chronic inflammation characterized by an elevated granulocyte activity and thus, an increase in the number of blood neutrophils. Colony-stimulating factors (CSF) constitute a group of four different cytokines (granulocyte-macrophage colony-stimulating factor (GM-CSF), macrophage colony-stimulating factor (M-CSF), granulocyte colony-stimulating factor (G-CSF) and multi-CSF (interleukin [IL-3]) [71]. They are produced by activated leukocytes and stimulate the proliferation, differentiation, maturation, and survival of granulocytes and macrophages. These cytokines play a myriad of roles in inflammation as pro-inflammatory cytokines [72,73,74]. The cytokine G-CSF regulates granulopoiesis, serves as a key mediator of the stress granulopoiesis response [75], and also affects neutrophil phenotype and function [76]. The G-CSF concentration increases in the serum at inflammatory sites [77,78]. Beneath its function as a CSF [75], GM-CSF affects and modulates more myeloid cell types, especially macrophages, granulocytes and eosinophils [79]. Expression of GM-CSF is normal under homeostatic conditions but increased under inflammatory conditions [80] as confirmed by the findings of this study.

Hamilton et al. [72] described a pro-inflammatory ‘CSF network’ of mutual dependence between CSF activity and the activity of monocyte- or macrophage-derived pro-inflammatory cytokines (such as IL-1 and TNF) [81].

In oral epithelial cells, IL-1b and TNF-alpha induce COX-2 leading to increased prostaglandin E2 production in HGFs [82,83]. In their study, Noguchi et al. [84] observed that high prostaglandin concentrations resulted in increased cytokine secretion, mainly due to positive feedback loops which contribute to the exacerbation of inflammation. In another report, De Oliveira et al. [85] stated that TNF-alpha plays a crucial role in the innate response against the periodontopathogenic bacteria. Interestingly, high levels of TNF-a, which activates pathways that culminate in the destruction of periodontal connective tissue and alveolar bone resorption [86,87], were detected in diseased sites of individuals with severe periodontitis [88].

The hepatocyte growth factor (HGF) is produced by fibroblasts from human gingiva by IL-1, TNF-alpha, and by prostaglandin E2 [89,90]. Other than that, it can be induced in culture by fimbriae of *P. gingivalis* [91] as well as *P. intermedia* [92]. This cytokine promotes the progression of periodontitis, by stimulating intense growth of epithelial cells and preventing regeneration of connective tissue attachments [93]. Increased levels of HGF in NG patients underline the pathogenic effects of HGF as a clinical parameter of disease progression [94].

IL-13 is a Th2 anti-inflammatory cytokine that regulates the collagen homeostasis by up-regulation of TGF-β [95] and down-regulation of collagen-destroying MMP-1 production [96]. Elevated levels of IL-13 have been detected in T-cells of severe periodontitis patients [97]. More recent research attributes IL-13 a pivotal role in the regulation of type 2 cytokine-mediated immune responses as a key inducer of many pathological processes [98].

The chemokine MIG (monokine induced by interferon (IFN)-γ) is stimulated by increasing production of IFN-γ and TNF-a from recruited Th1 lymphocytes, leading to a positive amplification feedback loop in inflammatory lesions [99,100]. MIG is considered as a marker of the host immune response due to its T-cell chemoattractant effect, has a central function in the recruitment of inflammatory cells [99,101], and is up-regulated by RANKL in osteoclast precursor cells [102]. The findings in our study are in agreement with past studies, which showed increased serum levels of MIG in different organs in autoimmune diseases [103].

The integration of the different data sources ties together the observations discussed so far at the microbial and protein levels. All significant inflammatory activities identified by the ELISA assay represent, together with the species highly abundant in the NG samples, a co-signature for the NG. In particular, a broader inflammatory response involving, together with the significantly overexpressed HGF and G-CSF clearly indicates that the shift in the microbial composition of the plaque associated with the transition from the milder CG to the more serious NG plays a relevant role, particularly via the *Campylobacter* spp., in the evolution of the most severe symptoms of the disease.

## 5. Conclusions

To the best of our knowledge, this study is the first attempt to detect inflammation markers in NG patients and to integrate the microbial and inflammatory landscapes of the disease. This allowed a better understanding of the complexity of the necrotizing disease in the context of cytokine production and pointed to the potential role of specific microorganisms in the orchestration of such inflammatory response.

In conclusion, this study indicates that NG is an opportunistic, mixed microbial infection with elevated cytokine levels in the serum. Our results suggest an association between specific microbial sub-ecosystems and elevated pro-inflammatory proteins in the blood serum. It is hard to gauge to what extent this represents a causal relationship. However, our findings further reinforce the theory of dysbiosis, i.e., that neither a specific periodontal pathogen nor cytokine is responsible for the disease, but the synergistic interaction of the microbial composition in combination with a diminished host response [62].

Patients undergoing preimplant surgical procedures are prone to contamination which can lead to perimplantitis [104,105]. Minimally invasive tools, such as digital dentistry can help to avoid microbial infections. Other approaches involve the use of implants with reduced bacterial leakage and emerging stem cell therapy [106,107].

Additional approaches, such as proteomic analysis, might help to further elucidate the molecular mechanisms underpinning the deteriorating pattern of periodontal diseases.

## Figures and Tables

**Table 1:** Characteristics of 16S rRNA-directed oligonucleotide probes used for FISH; target organisms, rRNA sequences, target site, and formamide concentration (F).

Probe ^1^	Target	Sequence (5′–3) ^2^	5′ modification	Target Site	F (%)	Source
BAC303	Most Bacteroidaceae and Prevotellaceae, some Porphyromonadaceae	CCA ATG TGG GGG ACC TT	Cy3	303–319	50	[45]
CFB935	Bacteroidetes (very broad)	CCA CAT GTT CCT CCG CTT GT	Cy3	935–954	50	[46]
EUB338	Many but not all bacteria/most eubacteria	GCT GCC TCC CGT AGG AGT	Carboxyfluorescein	338–355	40–50	[47]
L-Pgin1006–2	*P. gingivalis*	GTT TTC ACC ATC M**G**T CA**T** C	Cy3	1006–1024	45	[48]
Pend740	*P. endodontalis*	CAG TGT CAG ACG GAG CCT	Cy3	740–757	40	[49]
PRV392	*Prevotellaceae* (*Prevotella*, *Hallella*)/*Prevotellaceae* (*Prevotella* spp., *Alloprevotella* spp., *Hallella* spp.)	GCA CGC TAC TTG GCT GG	Cy3	392–308	50	[50]
Tfor127	*T. forsythia*	CTC TGT TGC GGG CAG GTT AC	Cy3	127–146	40	[33]

^1^ Probes were labeled at the 5′-end with Cy3 (Cyanine dye 3) or carboxyfluorescein. The designations of probes containing locked-nucleic-acid (LNA) substitutions start with L-. ^2^ Characters printed in bold indicate LNA substitutions. LNA incorporated DNA probes (LNA/DNA probes) have been described to significantly improve fluorescence intensity in comparison to conventional DNA probes with the same sequence.

## Data Availability

Reads pre-processing: The following string was specifically parsed to Trimmomatic: ILLUMINACLIP:adapters.fa:1:30:10 LEADING:5 TRAILING:5 SLIDINGWINDOW:5:15 AVGQUAL:30 HEADCROP:0 MINLEN:180; Reads alignment: The specific database used is from Silva v 138 and can be downloaded at https://zenodo.org/record/3986799#.X9j_bulKhTY, accessed on 14 August 2021), file *silva_species_assignment_v138.fa.gz*. Minimap was run with the following options: *-k 27 -p 0.5 -N 30 -x sr*. Krona Plots: Krona was run using the tool *ktImportText* on ad hoc formatted abundance files. Those were created by reconstructing the taxonomy of each species through the function *taxonomy()* from the R package myTAI.

## Supplementary Materials

The following are available online at https://www.mdpi.com/article/10.3390/antibiotics10101197/s1, Figure S1: Fluorescence microscopy (FM) panels illustrating the presence of specific phyla, genera and bacterial species related with necrotizing gingivitis. Images were generated with FISH and FM following hybridization with the following oligonucleotide probes: broad - spectrum CFB935 staining cells of the phylum *Bacteroidetes* with diverse morphology (A); BAC303 staining differently sized aggregates of *Bacteroides* spp., *Prevotella* spp., and *Porphyromonas* spp. (B); PRV392 staining rod - shaped cells of *Prevotella* spp. (C); Pend740 and l-Pgin1006-2 staining small, round cells of *P. endodontalis* (D) and *P. gingivalis* (E), respectively. The bacterial targets are zoomed and highlighted on white - framed subimages in panels A and C. Figure S2: Heatmap showing the concentration (in pg/mL) of a total of 30 cytokines, chemokines and growth factors among chronic gingivitis and necrotizing gingivitis patients. The rows are ordered by mean concentration value stratified according to the color bar on the right. Table S1: Characteristics of 16S rRNA-directed oligonucleotide probes used for FISH; target organisms, rRNA sequences, target site and formamide concentration (F).Table S2: Abundance and statistical analysis of the FISH-based quantification for the six selected bacterial probes. Table S3: Abundance and statistical analysis of the ELISA-based quantification for the thirty selected protein inflammatory markers.
