# Supplementary material for: Necrotizing Gingivitis: Microbial Diversity and Quantification of Protein Secretion in Necrotizing Gingivitis
Source: Antibiotics (Basel). 2021 Oct 1;10(10):1197. doi: 10.3390/antibiotics10101197 (PMC8532655; doi:10.3390/antibiotics10101197)
Supplement: Supplementary file 1 [file antibiotics-10-01197-s001.zip › antibiotics-1361523-supplementary/Supp_Table S1.pdf]

Supplementary Table S1

Characteristics of 16S rRNA-directed oligonucleotide probes used for FISH; target organisms, rRNA sequences, target site and formamide concentration (F)

Probe[1]	Target	Sequence (5' – 3)[2]	5' modification	Target Site	F (%)	Source
EUB338	most eubacteria	GCT GCC TCC CGT AGG AGT	Carboxyfluorescein	338 - 355	40 - 50	[11, 18, 19]
CFB935	Bacteroides-Porphyromonas-Prevotella subgroup of Bacteroidia	CCA CAT GTT CCT CCG CTT GT	Cy3	935 - 954	50	[17]
BAC303	Bacteroides-Prevotella subgroup of Bacteroidia	CCA ATG TGG GGG ACC TT	Cy3	303 - 319	50	[16]
PRV392	Prevotella spp.	GCA CGC TAC TTG GCT GG	Cy3	392 - 308	50	[22]
Pend740	*Porphyromonas endodontalis*	CAG TGT CAG ACG GAG CCT	Cy3	740 - 757	40	[21]
L-Pgin1006-2	*Porphyromonas gingivalis*	GTT TTC ACC ATC M**G**T CA**T** C	Cy3	1006 - 1024	45	[20]
Tfor127	*Tannerella forsythia*	CTC TGT TGC GGG CAG GTT AC	Cy3	127 - 146	40	[4]

[1]Probes were labeled at the 5'-end with Cy3 or carboxyfluorescein. The designations of probes containing locked-nucleic-acid (LNA) substitutions start with L-.

[2]Characters printed in bold indicate LNA substitutions. LNA incorporated DNA probes (LNA/DNA probes) have been described to improve significantly fluorescence intensity in comparison to conventional DNA probes with the same sequence [117].
