# Supplementary figures and images for: Necrotizing Gingivitis: Microbial Diversity and Quantification of Protein Secretion in Necrotizing Gingivitis
Source: Antibiotics (Basel). 2021 Oct 1;10(10):1197. doi: 10.3390/antibiotics10101197 (PMC8532655; doi:10.3390/antibiotics10101197)

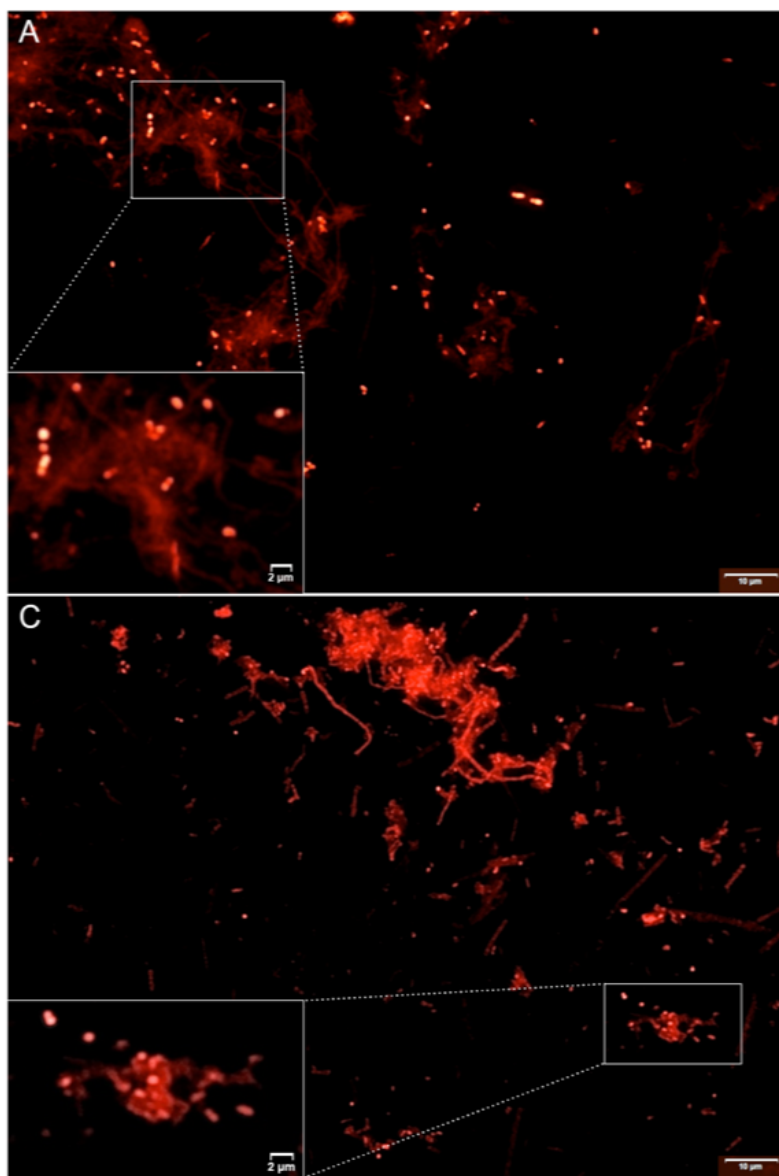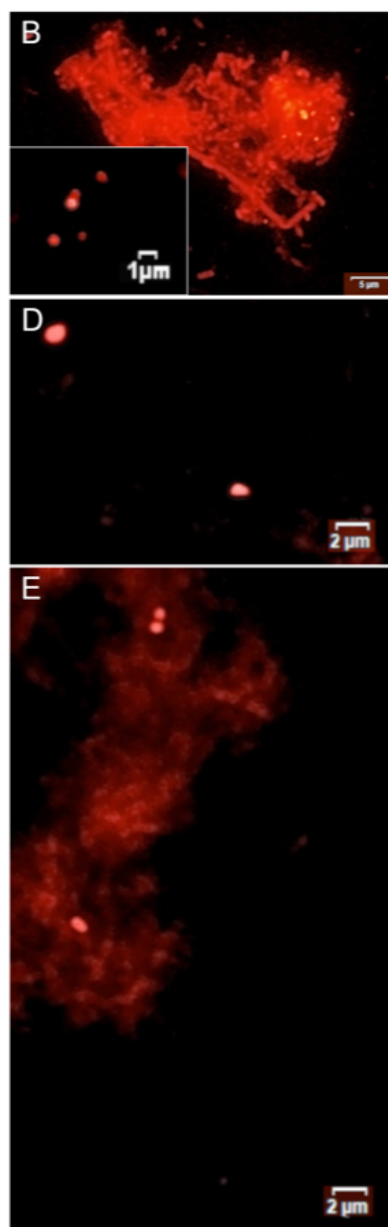

Supplement: Supplementary file 1 [file antibiotics-10-01197-s001.zip › antibiotics-1361523-supplementary/SuppFig_S1.pdf]

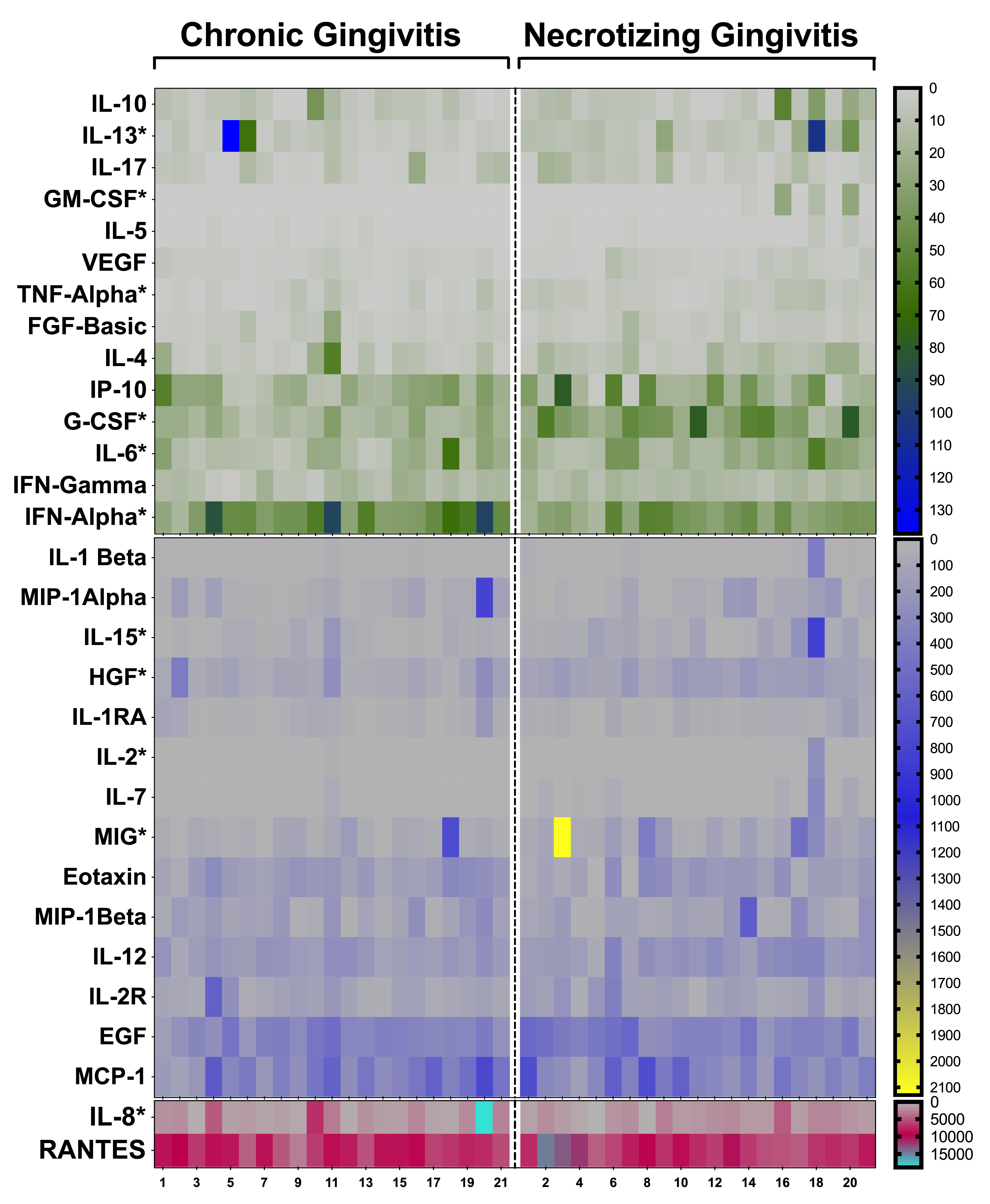

Supplement: Supplementary file 1 [file antibiotics-10-01197-s001.zip › antibiotics-1361523-supplementary/SuppFig_S2.tiff]
